# Supplementary material for: Mindfulness meditators show altered distributions of early and late neural activity markers of attention in a response inhibition task
Source: PLoS One. 2019 Aug 6;14(8):e0203096. doi: 10.1371/journal.pone.0203096 (PMC6684080; doi:10.1371/journal.pone.0203096)
Supplement: S1 File — Table A. Previous mindfulness research using the Go/Nogo task. Figure A. Source reconstruction during the well-known P100 occipital ERP, averaged across the 50 to 150 ms window across both groups using sLORETA and minimum norm imaging, unconstrained to cortex (to minimise assumptions). This was performed to demonstrate our source analysis was reliable even in the absence of individual MRI templates [61]. Note that the average does not depict positive or negative voltages, just whether a region was activated. Figure B. Single electrode ERP waveforms for both groups and conditions. These are depicted for comparison with traditional ERP analyses. Note that no statistics have examined data from single electrodes. (DOCX) [file pone.0203096.s001.docx]

**Supplementary Materials**

The following is a summary of previous research examining mindfulness with EEG activity during the Go/Nogo task. Six studies have used the Go/Nogo task to examine the effect of trait mindfulness or mindfulness meditation on ERPs related to conflict monitoring, response inhibition, and sustained attention (see Table A for a summary). Each has studied a different population or intervention, and results between studies are inconsistent (32-37). Studies have included healthy university students trained in one week of mindful deep breathing (32), adult ADHD and depression provided with MBCT for twelve weeks (33-35), healthy adolescents trained in eight weeks of the .b foundations mindfulness program (36), and healthy university students measuring the relationship between trait mindfulness and neural activity (37). All studies showed larger N2 amplitudes in the mindful participants, suggesting conflict monitoring or response inhibition processes. However, each study showed N2 alterations to different trial types, with (32) showing the increase in infrequent Nogo trials (and only in the 5 minutes / day condition), (36) to frequent Nogo trials, and (33) to frequent Go trials (but not Nogo trials), and (37) to both trial types (in individuals showing higher trait mindfulness). This suggests inconsistency in the relationship between neural changes resulting from mindfulness and conflict monitoring (low frequency trials) and response inhibition processes (Nogo trials). In addition to the inconsistency regarding N2 changes, only Schoenberg et al and Quaglia et al (33, 37) showed increased Nogo P3, suggesting enhanced response inhibition closure in more mindful participants. No study showed overall P3 changes (related to sustained attention), which we would expect from mindfulness meditation (which effects sustained attention).

**Table A. Previous mindfulness research using the Go/Nogo task.**

| Authors | Participants | Task design | Measures | Results |
| --- | --- | --- | --- | --- |
| Schoenberg and Spekens (2014a, b) | 51 adults with Depression, 26 undergoing 8 week MBCT, 25 WL control | 20% Nogo | FCz, Pz  Alpha, gamma and FMT activity 0-400 ms and 400-800 ms, and coherence between wide range of electrode pairs | MBCT reduced alpha power, which related to depression reduction. Alpha reduced particularly in response to negative material, and increased left fronto-parietal alpha coherence.  Also enhanced induced FMT post MBCT, correlated to reduced depressive and rumination symptoms. |
| Schoenberg et al (2014) | 26 ADHD adults, 24 waitlist control ADHD adults trained in twelve weeks of MBCT | 20% Nogo | Fz, FCz, Cz, Pz    P3, N2 (in both go and nogo trials) | Increased Nogo P3 at Pz (related to reduced impulsivity and inattention). Trend towards increased Nogo N2 in control group, but not in meditation group, who showed increased Go N2 at FCz. |
| Cheng et al. (2017) | Healthy control university students trained in five (N = 12), seven (N = 13), or nine (N = 13) minutes of deep breathing meditation, compared to controls (N = 13) | 20% Nogo | Fz, Cz, and Pz  P3, N2 (in both go and nogo trials) | Increased Nogo N2 amplitudes at all electrodes in 5 min group, which were larger than controls at endpoint. No changes to P3 in the five minute group, or the N2 in the seven and nine minute groups. |
| Quaglia et al (2015) | 62 healthy control university students measured in trait mindfulness | 30% Nogo, happy, neutral and fearful faces used as stimuli | N2 at FCz, P3 at Pz, and N1 at Cz | Trait mindfulness predicted larger N1 and N2 amplitudes in both Go and Nogo trials, as well as a more positive Nogo P3 (but not Go). 5-10% of variance in ERPs predicted by trait mindfulness. |
| Sanger et al. (2016) | 19 adolescents in eight weeks of mindfulness compared to 16 waitlist controls. | Oddball task design, 70% frequent Nogos, 10% target responses, and two infrequent Nogo conditions (10% each, one similar to the target but in a different colour, one different in shape to elicit P3a). | N2: AFz, Fz, F3 and F4,  P3a: Cz, C4, CPz, CP2, and CP4,  P3b: CP1, CP2, Cz, and CPz | Mindfulness group showed larger N2 to frequent Nogo trials, and trend towards larger N2 amplitudes to infrequency colour deviant Nogo at F4, which correlated with certain aspects of meta-cognitive awareness, as well as correlating with a decrease in perceived mental uncontrollability and improved cognitive confidence. |
| Sibalis et al. (2017) | 34 ADHD adolescents trained in 20 weeks of a “Mindfulness Martial Arts ” (similar to MBCT) and 22 waitlist controls | 33% Nogo trials | Theta:Beta ratios at Cz | Mindfulness group showed a reduction in the excessive theta and low beta ratios typical to ADHD, while control participants showed an increase in these ratios. |

**Supplementary Methods**

**Statistical methods**

RAGU’s GFP test uses the spatial standard deviation of the electric field to compare the global strength of cortical activation (42). The TANOVA assesses the percentage of randomly shuffled data sets that show larger scalp field differences between groups / conditions than the real data to determine whether to accept / reject the null hypothesis at a predetermined alpha level (42). Prior to the TANOVA, a Topographical Consistency Test (TCT) was conducted, comparing global field power within each group / condition to randomly shuffled data to ensure a consistent distribution of scalp activity within each group / condition. A significant TCT test confirms that any potential differences in the TANOVA are due to actual group / condition differences, rather than simply high variation within one of the groups (94). Because each of these tests only use a single value for comparison between groups / conditions at each time point (the spatial standard deviation for the GFP test, and the scalp field difference for the TANOVA and TCT test), no controls for multiple comparisons in the spatial dimension are needed even though all electrodes are included. To control for multiple comparisons in time (which are made at each time point in the epoch), global duration statistics calculate the duration of significant effects that are longer than 95% of the significant periods in the randomised data, ensuring significant durations in the real data last longer than the random comparison data at p = 0.05 (42). Additionally, global count statistics and area under the curve statistics of significant time points were checked to confirm sufficient control for multiple comparisons in the time dimension. The recommended L2 normalisation of scalp field variance across sensors was administered to remove scale differences, so that significant results in the TANOVA reflect a different distribution of neural activity without being affected by amplitude (42).

**Supplementary Results**


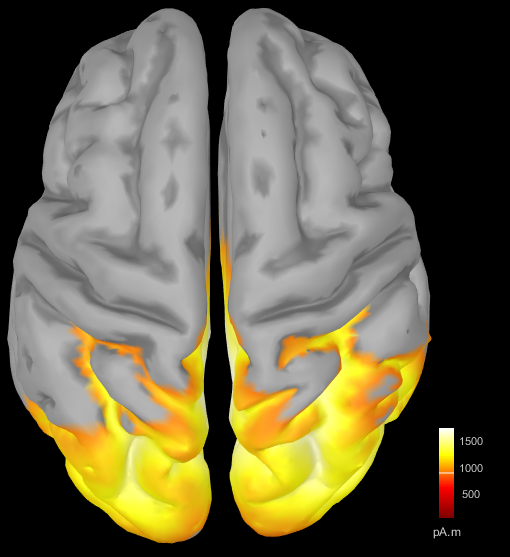


**Figure A. Source reconstruction during the well-known P100 occipital ERP, averaged across the 50 to 150 ms window across both groups using sLORETA and minimum norm imaging, unconstrained to cortex (to minimise assumptions).** This was performed to demonstrate our source analysis was reliable even in the absence of individual MRI templates (61). Note that the average does not depict positive or negative voltages, just whether a region was activated.


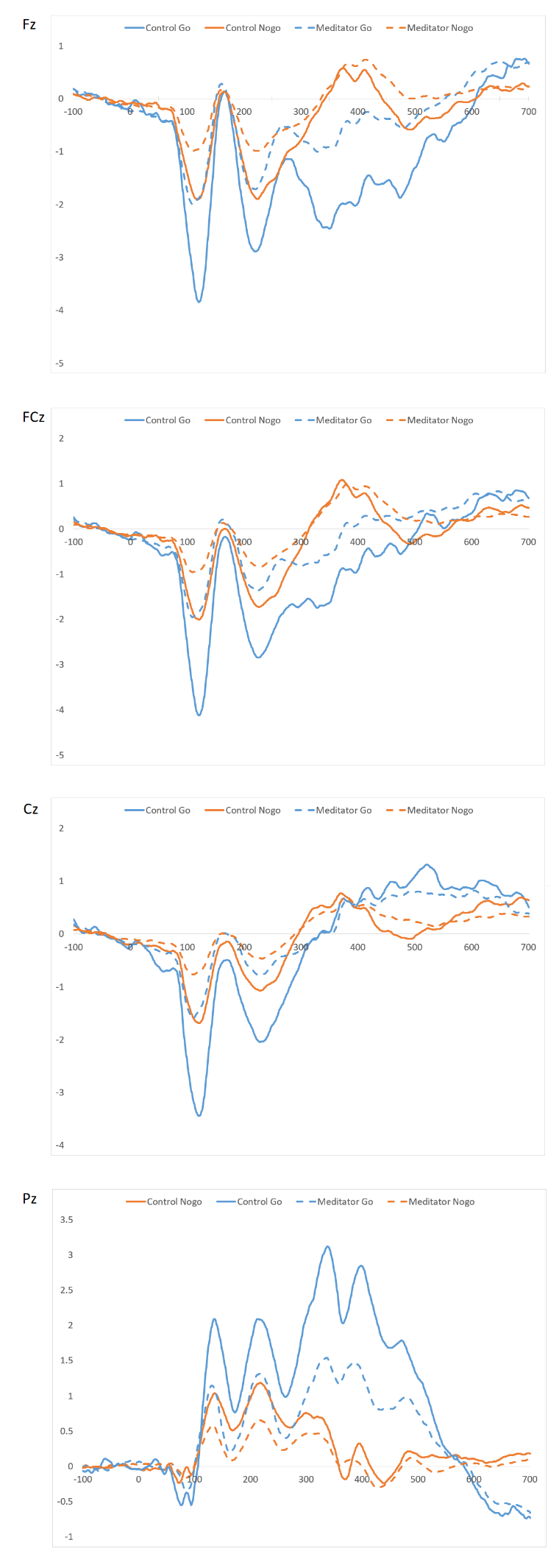


**Figure B.** **Single electrode ERP waveforms for both groups and conditions.** These are depicted for comparison with traditional ERP analyses. Note that no statistics have examined data from single electrodes.

**Supplementary Discussion Points**

Anticipatory activity has been found in the dorsal visual processing stream (in temporo-occipital regions), with top-down influences from lateral parietal attentional gating regions, and frontal control regions (68, 74, 78). These regions are similar to those shown in our source analysis. Additionally, attentional manipulations have been found from 0 to 50 ms time locked to stimuli presentation (89). Previous research examining these anticipatory brain responses and our current results cumulatively indicate that differences were not present in the primary visual cortex, suggesting attentional mechanisms modulating sensory processing, rather than altered initial sensory processing (78). These areas may exert an excitatory effect on primary visual areas that increase and prolong stimulus processing in those areas (79).

Additionally, the right occipital and temporal regions have been shown to specialise in processing faces as well as for anticipation of general visual processing, suggesting that higher activity in these regions in the meditation group is likely to assist stimuli processing in the current task (78, 80). As such, the results could reflect enhancement of the visual processing pathway so the chain of information from perception to performance is more effective (68). However, if the enhanced anticipation explanations for the altered pre-C1 topography are accurate, it is not clear why the difference detected in the pre-C1 period did not lead to alterations during the C1 and later sensory processing ERPs (despite being related to improved behavioural performance and enhanced theta synchronization to stimuli during the P3). We suspect neural activity for the time spanning the pre-C1 period to the P3 (including the C1, P1, N1, P2, and N2) must differ between meditators and controls, but in a manner too subtle to detect using ERP analyses (perhaps reflected by frequency or connectivity changes, or in action potentials too small to be detected at the scalp level). Future research may able to offer further insights.

The pre-C1 differences also raise a question. If the pre-C1 differences reflect altered anticipatory activity in meditators, do the results conflict with the conception of mindfulness as “being in the present moment”? Anticipatory activity necessitates processing ahead of the present moment. It may be that the concept of “being in the present moment” is a higher order neuropsychological processes, while automatic processes such as the C1 may still be engaged to anticipate stimuli, even as a result of the higher order processes resting “in the present moment”. In this explanation, non-judgemental awareness may apply not to discrete moments as measured with the millisecond precision of ERPs, but across the second or so that we feel subjectively aware, such that expectation for a stimuli within that second is “seeing reality as it is”. In which case, the conflict is between conceptual levels of processing, and essentially reflects a technical conflict. An alternative explanation is that the altered pre-C1 reflects lack of expectation, allowing neural processes during the pre-C1 window to be more available for processing the stimuli. Or one last alternative - activity in this region is simply strengthened in general in meditators, and as such the pre-C1 differences do not reflect anticipation or reaction to the stimuli, but just that the area is more prone to being active. However, the area was not activated more during other periods of the epoch, so the initial explanations seem more sensible to us.

An interesting and potentially useful feature of C1 activity is that, due to the anatomical structure of the primary visual cortex, the C1 polarity reverses depending on whether visual stimuli are presented in the upper or lower visual field (40). Future research could determine whether the altered pre-C1 in meditators reflects anticipation by presenting a visual processing task to meditators frequently in the lower visual field, and unexpectedly and infrequently in the upper visual field. If the effect of meditation on the pre-C1 reflects increased anticipation, unexpected stimuli may show a disruption of stimulus processing reflected by a reduced C1 compared to controls. In contrast, if the effect is a result of lack of expectation, the unexpected change in stimulus location will engage the C1 more quickly or strongly in meditators than controls (similar to the task tested by Kelly et al (40)).

### **Comparisons with previous research**

Our results were different to previous research using the Go/Nogo task. Previous research has shown increased amplitude of Nogo P3 (33) and increased Nogo N2 (32, 36), or decreased Nogo N2 (33). The current research showed less differentiation between P3 amplitudes to Go and Nogo trials in the meditation group, and a more frontal P3 topography. However, as mentioned, previous research used a lower proportion of Nogo trials, setting up an increased prepotent response tendency, likely exerting different task demands and different neural weak points for attentional improvements in the meditation group to focus upon. Additionally, while the current research found topographical differences during the pre-C1 and P3 period, previous research performed analyses using single electrode analyses, so could not discriminate between topography and amplitude changes, and during specified windows not including the pre-C1 period. Lastly, previous research examined less experienced meditators as participants, and included ADHD (33, 95), depressed (34, 35), and adolescent participants (36), so would have obtained smaller, perhaps less consistent and stable differences between groups, perhaps reflecting more short term changes to brain activity rather than permanent trait-like changes, and differences reflecting different preceding neural profiles of clinical and younger populations (for example reversal of the typical excess theta reduced beta ratio in ADHD (95)). Also, as mentioned in the introduction, the previous research is inconsistent.

More broadly, the current results align with a plethora of research indicating enhanced attention in mindfulness meditators. These studies have also shown that multiple aspects of attentional function are improved, including sustained attention (10-12), distribution of scarce attentional resources in time (13, 14) and space (15), and attentional control including inhibition of prepotent behaviour (11, 16, 88).

Lastly, as well as discriminating differences in strength of neural response from distribution, no previous research has examined the consistency of neural activity within meditation groups. Given the variability in practice, individuals, and other unknown factors, differences between meditation and control groups may in fact reflect simply differences in the degree of variability between groups. Traditional parametric statistics cannot account for within-group consistency (42). Demonstrating consistent within-group activity for meditators is an important step in ensuring the neural changes are practice-induced, and that the changes are common across the group. While not feasible to study longitudinally, prolonged meditation practice over many years is likely to result in the most consistent, durable and significant effects, and studying these individuals is likely to provide stronger conclusions about the effects of mindfulness meditation (9). As such, the consistent neural activity within groups in the current research is a strength of the study.

**Additional Potential Limitations**

It is important to note that potential explanations for the function of the altered mechanisms in meditators are based on correlational research. As such, we cannot be certain that the mechanism performs the suggested function, simply that it is related to it. A demonstrative example is that although the strongest correlation with hippocampal theta in rats is walking behaviour, complete lesioning of the hippocampus does not alter walking behaviour (96).

Because the pre-C1 effect was unexpected and has not been shown by prior research, we are less confident that it reflects a real difference between groups. However, it did show a large effect size and was highly significantly related to performance. The result should be replicated and explored further by future research, in order to confirm and explain the finding in more detail.

Another limitation is that our results may not generalise to clinical populations in which mindfulness is most commonly used - the mechanism of action in treatment interventions may be different. While mindfulness meditation is likely to alter attentional mechanisms in clinical groups, it may be that the practice does not alter the same mechanisms in those individuals as it does in healthy controls. As per our integrated interpretation of our results, attentional training is likely to alter neural activity where alterations are most necessary to improve function. These may be different in clinical and healthy populations. For example, depressed participants show altered Go/Nogo N2 activity compared to controls (46), so it may show activity in the N2 window that is altered in depressed individuals who practice mindfulness meditation.

Lastly, it should also be noted that although the language used in this study may suggest an agency behind attentional function, attention is not a homunculus guiding neural activation to achieve the goals it has set. The psychological concept of attention *is* the action of the neural activity, and other neural processes self-organising to achieve “goals”, which are themselves also simply behavioural reflections of neural processes. The origins of consciousness have not yet been explained.
